# Recent Advances in Liver Engineering With Decellularized Scaffold

**DOI:** 10.3389/fbioe.2022.831477

**Published:** 2022-02-10

**Authors:** Qingqing Dai, Wei Jiang, Fan Huang, Fei Song, Jiqian Zhang, Hongchuan Zhao

**Affiliations:** ^1^ Department of Hepatopancreatobiliary Surgery and Organ Transplantation Center, Department of General Surgery, The First Affiliated Hospital of Anhui Medical University, Hefei, China; ^2^ Department of Internal Medicine IV (Gastroenterology, Hepatology, and Infectious Diseases), Jena University Hospital, Jena, Germany; ^3^ Department of Burns, The First Affiliated Hospital of Anhui Medical University, Hefei, China; ^4^ Department of Urology, Jena University Hospital, Jena, Germany; ^5^ Department of Anesthesiology, The First Affiliated Hospital of Anhui Medical University, Hefei, China

**Keywords:** liver engineering, scaffold, decellularization, recellularization, implantation

## Abstract

Liver transplantation is currently the only effective treatment for patients with end-stage liver disease; however, donor liver scarcity is a notable concern. As a result, extensive endeavors have been made to diversify the source of donor livers. For example, the use of a decellularized scaffold in liver engineering has gained considerable attention in recent years. The decellularized scaffold preserves the original orchestral structure and bioactive chemicals of the liver, and has the potential to create a *de novo* liver that is fit for transplantation after recellularization. The structure of the liver and hepatic extracellular matrix, decellularization, recellularization, and recent developments are discussed in this review. Additionally, the criteria for assessment and major obstacles in using a decellularized scaffold are covered in detail.

## 1 Introduction

Globally, end-stage liver disease is a primary cause of morbidity and mortality, accounting for more than one million deaths per year (Cheemerla and Balakrishnan, 2021). End-stage liver disease is a growing public health hazard for which liver transplantation is the only available remedy.

Since Thomas Starzl conducted the first liver transplantation in 1963, liver transplantation has evolved significantly over the last 50 years. Along with the renewal of immunosuppressive drugs, liver transplantation technology has increasingly advanced, resulting in the survival of numerous patients with end-stage liver disease (Song et al., 2014). Unfortunately, donor scarcity continues to be a significant issue. Aside from increasing citizen donation rates and marginal liver utilization, efforts are required to develop alternative viable treatments (Muller et al., 2020; Yagi et al., 2020).

**FIGURE 1:**
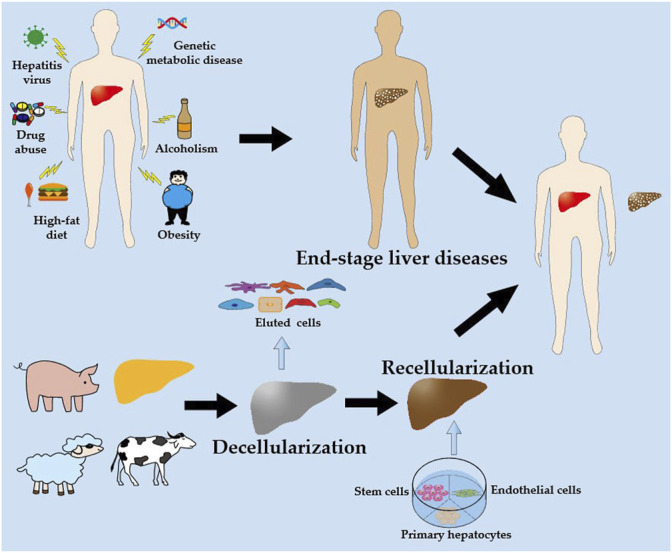
A schematic illustration of the liver engineering based on decellularized scaffold. Livers obtained from xenogeneic animals (such as pigs, sheep and cattle) and discarded human livers are decellularized to produce decellularized scaffolds. The decellularized scaffolds are then recellularized with hepatocytes, endothelial cells, and stem cells, which are perfused *in vitro* to obtain a new liver. Ultimately, the new liver is transplanted into the patient with end-stage liver disease to replace the original decompensated liver.

In recent years, the use of decellularized scaffolds in liver engineering has garnered considerable attention (Figure 1). Decellularized scaffolds from xenogeneic animals (such as pigs, sheep, and cattle) and discarded human livers preserve the multifaceted extracellular matrix (ECM) of the liver and serve as a reservoir for growth factors, cytokines, and signaling molecules that are critical and indispensable for cellular growth, proliferation, differentiation, and neovascularization (Choudhury et al., 2020). Decellularized scaffolds are subsequently repopulated with primary hepatocytes, endothelial cells, and stem cells, which ultimately develop new livers that can be transplanted into patients with end-stage liver disease to replace the original decompensated livers. Increasing evidence suggests that decellularized scaffolds can sustain implanted cells for extended periods, enabling subsequent implantation (Kakabadze et al., 2018; Yang et al., 2018; Liu et al., 2019; Everwien et al., 2020b; Shaheen et al., 2020).

Therefore, the recellularization of decellularized scaffolds holds great therapeutic potential for creating transplantable grafts for treating end-stage liver disease. This review provides an overview of the liver, hepatic ECM, decellularization, recellularization, and recent advances in liver engineering. Additionally, the review presents the detailed evaluation criteria and main obstacles for utilizing decellularized scaffolds.

## 2 Liver and Hepatic ECM

### 2.1 Liver Structure

The liver is the largest internal organ in the body and is composed of various cell types, including hepatocytes, liver sinusoidal endothelial cells (LSECs), Kupffer cells, stellate cells, and cholangiocytes (Gordillo et al., 2015; Trefts et al., 2017; Stamataki and Swadling, 2020). These cells function in tandem to regulate liver function.

**FIGURE 2:**
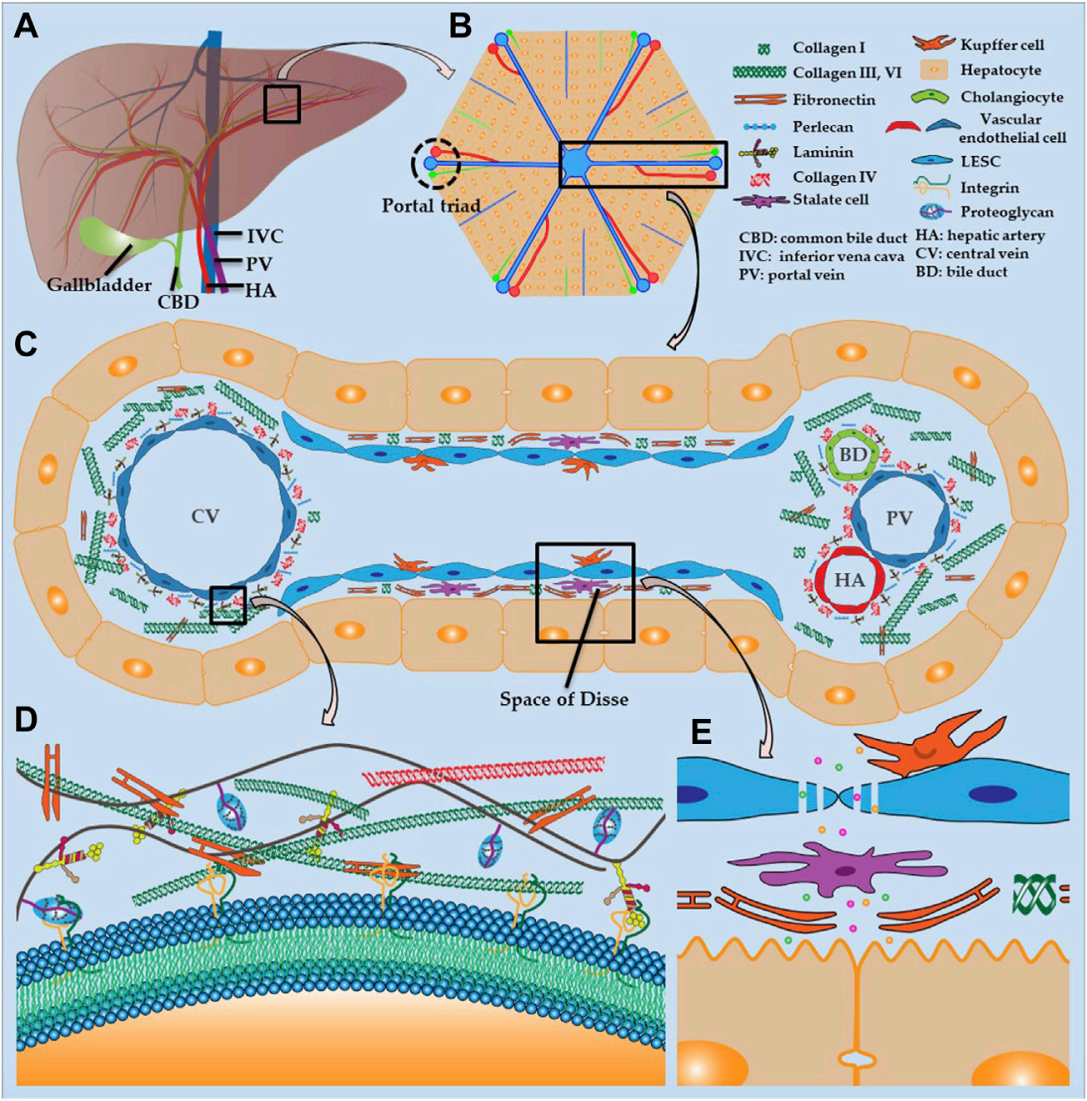
Liver structure and hepatic ECM **(A)** Distribution of hepatic vasculature and bile ducts **(B)** Histology of liver lobule **(C)** Composition of hepatic sinusoids and ECM **(D)** ECM structure **(E)** Fenestration of LSECs and structure of space of Disse.

The liver receives a dual blood supply from the hepatic artery (HA) and portal vein (PV), and transports the bile that is synthesized by hepatocytes to the gallbladder and duodenum *via* the common bile duct (Figure 2A). The liver lobule (Figure 2B) is the anatomical structural unit of the liver and comprises hexagonal cords of hepatocytes, which are the principal parenchymal cells of the liver; these cells account for approximately 80% of the cellular composition of the liver. The central vein is located in the middle of each liver lobule, while the portal triad is located on the periphery and is composed of closely bundled branches of the HA, PV, and bile duct.

The hepatic sinusoid is a capillary-like channel lined with LSECs and flanked by hepatocyte plates and drains blood from the terminal branches of HA and PV into the central vein (Figure 2C) (McQuitty et al., 2020). Kupffer cells are resident macrophages that are localized in the hepatic sinusoid and adhere to LSECs. These cells are responsible for the uptake and destruction of foreign materials that are transported to the liver, such as bacteria, endotoxins, and microbial debris. The sinusoidal endothelium is primarily composed of LSECs, which are highly specialized endothelial cells and the most abundant non-parenchymal cells in the liver. These cells are located at the interface between the liver parenchyma and circulatory system (Poisson et al., 2017; Gracia-Sancho et al., 2021). Unlike other endothelial cells, LSECs have a unique phenotype because they lack the basement membrane and have a profusion of fenestrations (Figure 2E) (Li et al., 2020). Consequently, LSEC fenestrations and slow blood flow in the hepatic sinusoid enable hepatocytes to exchange sufficient substances with the blood in the space of Disse to perform essential functions, such as the uptake of toxins for processing and the secretion of synthesized serum proteins. Stellate cells are present in the space of Disse and allow the liver to respond to injury and heal certain types of damage. The surface of hepatocytes, which face the hepatic sinusoid, consists of a basolateral membrane with many microvilli, which dramatically increase the contact area between hepatocytes and plasma (Figure 2E). The smooth lateral membrane connects to adjacent hepatocytes and forms a narrow, tubular channel, which drains toxins, bile, and other digestive molecules secreted by hepatocytes to the bile duct. Cholangiocytes are epithelial cells of the bile duct that release bicarbonate and water, which contribute to bile secretion.

### 2.2 Hepatic ECM

The hepatic ECM is a well-organized non-cellular tridimensional scaffold composed of collagens, proteoglycan, fibronectin, laminin and several other glycoproteins, all secreted by resident cells in liver, and provides a microenvironment for adhesion, growth, replication, migration, differentiation, and other vital activities of the surrounding cells (Figure 2D). The hepatic ECM consists of the interstitial matrix, which fills in the intercellular space, and a basement membrane, which separates the endothelium from the underlying connective tissue. The basement membrane, which is composed of laminin, collagen type IV, and perlecan, is mainly distributed around the portal triad and central vein (Figure 2C) (Mak and Mei, 2017; Zhang et al., 2018). The liver parenchyma lacks a complete basement membrane, and the fibronectin and collagen I in the space of Disse separate the hepatocytes and hepatic sinusoids. The interstitial matrix comprises collagen types I, III, VI, and fibronectin, which provides a place for cells to attach and reside, and buffers against compression that is placed on the liver. Likewise, integrin is a transmembrane receptor that acts as a cement and modulator by binding to ligands, such as fibronectin, collagen, laminin, and cell surface proteins, and promotes cell-cell or cell-matrix adhesion and interaction (Figure 2D).

The composition of the hepatic ECM fluctuates with age. For example, older livers tend to have greater amounts of total collagen, sulfated glycosaminoglycans (GAGs), and lower levels of growth factors. Therefore, donor age should be considered as a critical component in determining the quality of the neo-liver during liver engineering (Acun et al., 2021).

## 3 Decellularization

Liver engineering is presently considered a promising alternative strategy for organ scarcity. Additionally, the use of synthetic and decellularized scaffolds in liver engineering has emerged as a research hotspot in liver engineering. The decellularized scaffold is more advantageous than the synthetic scaffold because it preserves the natural microscopic structures and microenvironment, retains bioactive molecules, and allows for implantation of the liver through directly anastomosing the native vessels of the scaffold and the vessels of the recipient (McCrary et al., 2020). Decellularization is the elimination of cellular components from native tissues or organs, while conserving the ECM and bioactive molecules. Physical methods, chemical reagents, and enzymatic treatments are commonly used for decellularization.

### 3.1 Physical Methods

#### 3.1.1 Sonication

Sonication uses an ultrasonic bath or probe to transfer acoustic power into a solvent containing tissues or organs to disrupt cell membranes; an ultrasonic bath is preferred for decellularization. The collapse of microscopic vacuum bubbles generated during sonication releases violent shockwaves that shear the cell; the resulting cellular debris is removed by perfusion. The energy generated from the collision of the sound waves generates heat in the solution. Therefore, monitoring the temperature during sonication and cooling in a chiller are necessary to avoid heating and denaturation of the proteins and molecules. Sonication is mainly used with detergents to decellularize dense tissues, such as tendons, ligaments, and cartilage, as well as thin tissues, such as skin and blood vessels, where sonication allows the detergents to penetrate the tissues more effectively (Yusof et al., 2019; Shen et al., 2020; Dang et al., 2021; Lin et al., 2021; Suss et al., 2021). Sonication has only been used to decellularize whole parenchymal organs in the kidney, and has likely not been used in the liver because of the colossal size of liver and the need for high power to disrupt the hepatic microstructure (Say et al., 2019; Manalastas et al., 2021).

#### 3.1.2 Freeze-Thaw

Rapid thermal changes in the freeze-thaw effectively lyse cells and aid in their subsequent removal (Tao et al., 2021). Hence, a single freeze-thaw cycle is commonly used as the first step to reduce the quantities of chemical reagents for liver decellularization (Cheng et al., 2019). However, a single freeze-thaw cycle can still impact the microstructure and mechanical properties of the ECM due to the formation of ice crystals (Cheng et al., 2019). As a result, some researchers have advocated the use of cryoprotectants in perfusion-mediated decellularization to mitigate the detrimental effects of the freeze-thaw cycle without affecting cell lysis. For example, Pulver reported that pretreatment with 5% trehalose achieved the same result as an overnight freeze-thaw cycle, but with lower microstructural damage (Pulver. et al., 2014).

#### 3.1.3 Immersion and Agitation

Immersing tissues or organs in a reagent is the most straightforward method of decellularization, and when paired with agitation, the outcomes of decellularization are improved. The reaction efficiency depends on the reagent type, duration of immersion, and intensity of agitation. A reagent for the removal of the cellular components is required, and the total duration should be minimized to avoid damaging the ECM scaffold (Mattei et al., 2014). However, this approach is usually only appropriate for epidermal tissues and smaller organs, such as the small intestine submucosa, blood vessels, trachea, cornea, nerve, and thyroid gland, and is not appropriate for larger parenchymal organs (Syed et al., 2014; Guimaraes et al., 2019; Visscher et al., 2021; Changchen et al., 2021; Weng et al., 2021). The required DNA content of 50 ng/mg in the decellularized tissue is only achieved when the thickness of the liver slices or discs is less than 5 mm (Mattei et al., 2014; Mazza et al., 2019).

### 3.2 Chemical Methods

#### 3.2.1 Detergents

Ionic, non-ionic, and zwitterionic detergents are commonly used for decellularization. The most extensively used ionic detergents are sodium dodecyl sulfate (SDS) and sodium deoxycholate (SDC), which solubilize cellular membranes and denature proteins (Zhang et al., 2018; Willemse et al., 2020; Felgendreff et al., 2021). Triton X-100 is the most widely used non-ionic detergent and is capable of breaking lipid-lipid, lipid-protein, and DNA-protein interactions. Triton X-100 is considered a gentler detergent than SDS. Furthermore, Jeong reported that SDS and SDC significantly disrupted GAGs and elastin, albeit more efficiently than Triton X-100 (Jeong et al., 2021). Additionally, the protocol using Triton X-100 alone conserved 1.5-fold more collagen and 2.5-fold more GAGs in the liver than that using Triton X-100 + SDS (Willemse et al., 2020). Zwitterionic detergents (such as 3-[(3-cholamidopropyl) dimethylammonio]-1-propanesulfonate (CHAPS)) exhibit both non-ionic and ionic characteristics, and are less harmful to proteins because of the net-zero electrical charge on the hydrophilic groups (O'Neill et al., 2013). Because of its low permeability, CHAPS is frequently used in thin tissues and organs, and its use in the liver has been rarely reported (O'Neill et al., 2013; Tajima et al., 2018; Shimoda et al., 2019).

Although diverse detergents are frequently combined for optimal decellularization, the efficiency of detergents nonetheless varies linearly with the exposure time and concentration, and varies inversely with the tissue thickness and density. Prolonged exposure times and high detergent concentrations can maximize the extent to which the ECM of the scaffold approaches the cell-free state. However, the ECM can also be disrupted and residual detergents may be present, particularly if SDS is used, which can penetrate deeply into the tissue and slowly dissipate. The disruption of the ECM and residual detergents may result in the formation of thrombi and cytotoxicity after recellularization and implantation, particularly in the liver because of its abundant blood flow and metabolic importance.

#### 3.2.2 Hypertonic and Hypotonic Solutions

Hypertonic and hypotonic solutions promote cell shrinkage and swelling due to osmosis and ultimately induce lysis, while having a negligible effect on the ECM (Kim et al., 2016). Although osmosis can kill cells, it is inefficient and must be coupled with detergents or enzymes to obliterate the cellular components in the liver (Kobes et al., 2018; Lewis et al., 2018). Joseph reported that the sequential treatment of mouse liver with a hypotonic solution and Triton X-100 resulted in a scaffold that was devoid of cells and maintained its structural integrity, as confirmed by the scanning electron microscopy (SEM) images (Kobes et al., 2018).

#### 3.2.3 Acids and Bases

Although acids remove DNA by dissolving cytoplasm and breaking nucleic acids during decellularization, they are more currently and popularly used as disinfectants because they are strong oxidants (Vishwakarma et al., 2019; Gao et al., 2020; Hussein et al., 2020). In a highly alkaline solution, double-stranded DNA denatures low-viscosity single-stranded DNA, which is easily removed by perfusion (Sengyoku et al., 2018). Relevant studies have demonstrated that a highly alkaline NaOH-PBS solution could achieve the same effect as SDS and CHAPS during decellularization while also enhancing biocompatibility and vascular regeneration of scaffolds (van Steenberghe et al., 2017; Sengyoku et al., 2018; van Steenberghe et al., 2018). Additionally, highly alkaline solutions with a pH of 12 or more can inactivate conventional bacteria and viruses, as well as unconventional prions, which are pathogens that are often associated with the use of biomaterials (van Steenberghe et al., 2017; Sengyoku et al., 2018). However, acids and bases can damage collagen and undermine protein-protein bonds, leading to microstructural destruction and decreased viscoelasticity (Reing et al., 2010). In addition, sodium hydroxide cleaves nearly all the growth factors, such as fibroblast growth factor and vascular endothelial growth factor (Reing et al., 2010).

#### 3.2.4 Alcohols

Because of the polar hydroxyl groups, alcohols can diffuse into cells and resultantly dehydrate and lyse cells (Flynn, 2010). Because alcohol is more efficient at removing lipids than lipase, alcohols (such as glycerol) are frequently used to extract lipids from adipose tissue (Flynn, 2010; Brown et al., 2011). Alcohols can precipitate proteins and destroy the ultrastructure of the ECM; therefore, care must be taken when they are used to remove cellular components. Compared to acids, alcohols are more routinely used to sterilize decellularized matrix materials. Ethanol (4%) and peracetic acid (0.1%) are frequently used to sterilize decellularized scaffolds in liver engineering (Xiang et al., 2016; Devalliere et al., 2018; Nishiguchi and Taguchi, 2021; Sassi et al., 2021).

#### 3.2.5 Chelates

Chelates (such as ethylenediaminetetraacetic acid (EDTA) and ethylene glycol tetraacetic acid (EGTA)) bind metallic ions that are essential for protein interaction, resulting in the disconnection of intercellular integral proteins and disruption of cellular adhesion in the ECM. However, chelates alone are unable to completely remove cells and should be combined with detergents or enzymes (Zhao C et al., 2020; Faccioli et al., 2020; Lorvellec et al., 2020). Additionally, using EDTA in the decellularization technique reduces the residual DNA and produces more intact ECM (Maghsoudlou et al., 2016).

### 3.3 Enzymatic Treatments

Nucleases, trypsin, dispase, lipase, and collagenase are commonly used in decellularization, whereas nucleases and trypsin are most frequently used in liver decellularization. Enzymes can precisely remove cellular components, prevent unfavorable immune responses, and improve the efficacy of detergent-based decellularization.

Nucleases are enzymes that cleave the phosphodiester bonds between nucleotides in nucleic acids. They typically expedite the removal of nucleotides and minimize the risk of immune responses during decellularization. Endonucleases, such as DNase and RNase, are frequently used to remove nucleic acids during liver decellularization (Ahmed et al., 2020; Everwien et al., 2020b; Wu et al., 2020). Trypsin is a serine protease that hydrolyzes the proteins involved in cellular attachment; consequently, trypsin dissociates cells from adhering tissue. The regular replacement of trypsin is necessary because protease inhibitors that are released from broken cells restrict trypsin activity after prolonged circulation (Prasertsung et al., 2008). Additionally, the enzymes can penetrate deeper into the tissue under pressurized conditions to increase the reaction between the enzyme and the substrate, which aids in cellular removal and shortens the reaction time (Prasertsung et al., 2008).

### 3.4 Combination

Each of the aforementioned methods (Sections 3.1–3.3) has advantages and disadvantages. Although physical methods are less disruptive to the microstructure of the tissue, they do not remove cellular components. Comparatively, although chemical agents can significantly remove cellular components, they inevitably destroy the microstructure and ECM. The preferred method of decellularization varies tremendously among different tissues and organs, and the ideal protocol should include at least two of the aforementioned methods to maximize their respective advantages and minimize the disruption of the ECM. Currently, the most frequently used protocol for liver decellularization is a freeze-thaw cycle followed by a combination of detergents, enzymes, and chelating agents (details presented in Table 1).

**TABLE 1:** Common methods and combinations used in liver decellularization.

Common methods	Advantages	Disadvantages
Freeze-thaw	Effectively disrupts cell membrane and lyses cell	Does not remove cellular components
	Less disruptive to ECM	Ice crystal formation disrupts microstructure of ECM and reduces mechanical strength
Detergents	Effectively lyse cells inside the organs	Disrupt proteins and microstructure of ECM
	Effectively remove cellular and nuclear components	Reduce the contents of growth factors
		Residual detergents cause thrombus and cytotoxicity
Chelates	Effectively disrupt cell adhesion to ECM	Do not actually lyse cells
	Reduce the residual DNA	Denature proteins of ECM
	Produce more intact ECM	Ineffective when used alone
Enzymes	Precisely remove cellular components	Regular replacement
	Reduce immune responses	Disrupt proteins and distort ECM structure

Common combinations	Overview	References
Freeze-thaw + detergents + chelates + enzymes	Free-thaw + Triton X-100 + EGTA + trypsin	Miyauchi et al. (2017)
		Minami et al. (2019)
		Takeishi et al. (2020)
	Free-thaw + Triton X-100/SDC + EDTA + trypsin	Coronado et al. (2017)
	Free-thaw + Triton X-100 + EDTA + trypsin	Faccioli et al. (2020)
Freeze-thaw + detergents + chelates	Free-thaw + Triton X-100/SDS + EGTA	Gao et al. (2020)
	Free-thaw + Triton X-100/SDS/CHAPS + EGTA	Shimoda et al. (2019)
	Free-thaw + Triton X-100/SDS/SDC + EDTA	Thanapirom et al. (2021)
Freeze-thaw + detergents + enzymes	Free-thaw + Triton X-100/SDS + DNase/RNase	Lu et al. (2018)
		Wu et al. (2020)
	Free-thaw + Triton X-100/SDS + DNase	Everwien et al. (2020b)
		Willemse et al. (2020)
	Free-thaw + SDS + DNase	Buhler et al. (2015)
Detergents + chelates + enzymes	SDC + EDTA + DNase	Lorvellec et al. (2020)
	Triton X-100 + EGTA + trypsin	Watanabe et al. (2019)
		Zhao C et al. (2020)
	Triton X-100 + EDTA + DNase	Caires-Junior et al. (2021)

Perfusion *via* the HA and PV is the preferred method for whole liver decellularization. To lyse and remove cells in the liver, detergents, enzymes, and other reagents are perfused into the vasculature of the liver; this approach ensures that the maximal integrity of the ECM is maintained. For example, decellularized liver contained less residual DNA, with a more homogeneous ECM, under oscillatory pressure and pressure-controlled perfusion; this approach also required a relatively short time, and more collagen and GAGs were retained in the decellularized liver (Struecker et al., 2017; Shaheen et al., 2020). Benjamin first reported that applying oscillatory pressure to rat livers that were perfused with 1% Triton X-100 and 1% SDS further enhanced microperfusion and the homogeneity of decellularization by simulating intra-abdominal conditions during respiration (Struecker et al., 2017). Shaheen noted that porcine livers decellularized by PV perfusion of 1x Triton X-100 and 0.6% SDS maintained a perfusion pressure of 8–12 mm Hg and preserved the parenchymal liver lobule structures when compared with the native liver (Shaheen et al., 2020).

### 3.5 Evaluation of Decellularized Liver ECM

The ultimate goal of decellularization is to remove all the cellular components while retaining an intact ECM for recellularization. However, no method has been able to satisfy this standard. Following recellularization, the remaining cellular components in the ECM create an unfavorable host immune response, and the deterioration of the ECM will also compromise the bioactivity of the implanted cells. Thus, establishing a quality standard for decellularization and scaffolds can facilitate the rapid and organized development of liver engineering and better satisfy the requirements for clinical applications.

#### 3.5.1 Evaluation of Decellularization Efficiency

Presently, the degree of decellularization is primarily determined by the residual DNA, which is ubiquitous in tissues and cells and is directly associated with the host immune response. The generally acknowledged criteria are as follows: 1) DNA content <50 ng/mg dry ECM weight; 2) DNA fragment length <200 base pairs; and 3) lack of visible nuclear content with DAPI and HE staining. Narciso et al. lately described a novel method that combines phase-contrast images of decellularized tissues and fluorescent images of DNA staining of cell nuclei, allowing faster and easier quantification of DNA content (Narciso et al., 2021). However, investigations have shown that the cytoplasm and components of the cell membrane can also cause immune responses and inflammation (Chakraborty et al., 2020). Therefore, these aforementioned criteria should be upgraded and modernized to ensure accuracy and uniformity.

Although the abovementioned criteria have been widely adopted, they all lead to destructive end-points in analyses of the scaffold, and the degree of decellularization cannot be determined in real-time. Consequently, it is difficult to tailor decellularization to the distinctive attributes of each liver. Therefore, it is essential to develop a new non-invasive method to monitor and adjust decellularization in real-time to achieve individualization (Hulsmann et al., 2015; Geerts et al., 2016).

Akhyari et al. employed rheological evaluation and biomass compositional analysis of the perfusate for the real-time and non-invasive monitoring of heart decellularization. They discovered that the total protein, DNA, and biomass content in the perfusate was proportional to its viscosity. Based on this finding, the decellularization protocol could be adapted for a single organ. Furthermore, this finding demonstrated that the adverse effects of reagents on the ECM could be minimized if the pump profile, perfusion pressure, and revolution speed in the feedback control system were controlled (Hulsmann et al., 2015). Geerts et al. found that the HU value in CT scans was positively correlated with the quantity of DNA remaining in the scaffold during liver decellularization. These results suggest that the residual DNA could be used to predict the extent of decellularization. This established methodology when combined with characterization of the perfusate could be optimized to create a well-preserved scaffold (Geerts et al., 2016).

#### 3.5.2 Quality Assessment of Decellularized Liver Scaffold

As the mold for liver engineering, the quality of the decellularized scaffold is crucial for successful liver recellularization and transplantation. However, there is currently no unified criteria for assessing the quality of the scaffold. The majority of qualitative and semi-quantitative studies have used morphological and molecular characteristics to assess the decellularized scaffold. The morphological assessment uses the appearance, HE staining, and SEM of the scaffold to evaluate its remaining microscopic anatomy and three-dimensional structure. The molecular assessment includes quantitative assessment of the residual DNA, cytokines, and ECM proteins. In addition, many studies have compared the mechanical strength, tension resistance, and other physical properties of the decellularized liver with those of the native liver (Everwien et al., 2020a).

Philipp et al. recently used macroscopic, microscopic, and morphological assessments to develop a morphological workflow for analyzing the microstructure of decellularized mouse liver scaffolds with mild histological damage (hepatic steatosis, hepatic fibrosis, and nodular regenerative hyperplasia) (details presented in Table 2). The results indicated that this procedure aided in evaluating the quality of decellularized scaffolds and in identifying the tissues with the best-preserved microstructure and matrix components; these results are also fundamental for future repopulation and transplantation trials (Felgendreff et al., 2021).

**TABLE 2:** The sequential morphological workflow to identify liver scaffold-sections with well-preserved microarchitecture.

—		Grading		Structure	Score
Macroscopic assessment		Good		Complete removal of hepatic tissue of the whole liver lobe	4
		Moderate		Incomplete removal of hepatic tissue in less than half of the parenchyma of a single liver lobe, predominantly at the edges of the liver lobes	3
		Limited		Incomplete removal of hepatic tissue in more than half of a single liver lobe	2
		Poor		No removal of hepatic tissue within one or more liver lobes	1
Microscopic assessment		The microscopic assessment was applied to all samples without macroscopically visible tissue remnants. Only sections without evidence of residual cell nuclei or cytoplasmic glycogen were subjected to further analysis		

—	—	**Parameter**	**Structure**	**Present**	**absent**
Morphological assessment	Histological score	Portal vein integrity	Presence of portal vein in HE staining	1	0
			Presence of continuous elastic fibers of the portal vein in EvG staining	1	0
		Central vein integrity	Presence of central vein in HE staining	1	0
			Presence of continuous elastic fibers of the central vein in EvG staining	1	0
		Sinusoidal network integrity	Presence of sinusoidal network	1	0
			If present, continuity of network structure*	1	0
	Immunohistochemical score	Portal vein integrity	Presence of collagen I and collagen IV signals in the portal vein	1	0
			Presence of continuous elastic fibers and basal membrane of the portal vein in elastin as well as laminin staining	1	0
		Central vein integrity	Presence of collagen I and collagen IV staining in the central vein	1	0
			Presence of continuous elastic fibers and basal membrane of the central vein in elastin as well as laminin staining	1	0
		Sinusoidal network integrity	Presence of sinusoidal network in collagen I and collagen IV staining	1	0
			Continuity of the sinusoidal network in collagen I and collagen IV staining, if present*	1	0

* Continuous: 1, interrupted: 0

However, morphological scoring methods vary depending on the expertise and subjectivity of the observer. Therefore, it is necessary to create digital image analysis to decrease this variance and achieved increased accuracy. Magliaro et al. developed a faster and more intuitive open-source image analysis software, HisTOOLogy, and applied it to analyze the effects of different decellularization protocols, showing a positive linear relationship between cell count and eosin staining area with total DNA and total protein quantification (Magliaro et al., 2015). The results demonstrated HisTOOLogy’s ability to analyze and provide quantitative information on histological sections, which aids in the efficiency and accuracy of evaluating decellularized scaffolds (Mattei et al., 2017). Besides, Moulisová et al. combined semiquantitative histological analysis with a newly developed software application (ScaffAn), which uses automated image analysis (details presented in Table 3) (Moulisova et al., 2020), to create a novel multi-scale morphological evaluation system for determining the quality of decellularized liver scaffolds. Comparatively, the conventional semi-quantitative histological analysis is based on seven criteria and a matching three-level grading system, which uses the morphological quality based on HE staining and SEM. In the quantitative analysis, the length of the sinusoidal structure and number of sinusoidal network branches are calculated based on high-resolution slide scans of the HE stains by using the newly developed software. Therefore, this approach complements the conventional analysis and effectively improves the discriminative power.

**TABLE 3:** Morphological multi-scale evaluation system.

	Method	Magnification	Parameter	Structure	Score
conventional semiquantitative assessment	HE staining	5×	Lobular shape	Compression to 1/3 of the original shape	0
				Compression to 2/3 of the original shape	1
				Preservation of lobular shape	2
	HE staining	5×	Sinusoidal network presence	Present in less than 50% of lobules	0
				Present in 50–90% of lobules	1
				Present in more than 90% of lobules	2
	HE staining	10×	Septa and triad structures	Destroyed septa and triad structures	0
				Ruptured septa and/or separation into layers	1
				No rupture, no separation of the septa into layers, vessels well defined in triads	2
	HE staining	15×	Sinusoidal network integrity	Large differences in distance between individual sinusoids	0
				Some network irregularities	1
				Regular distribution and consistent network structure	2
	SEM	2000×	Sinusoidal wall integrity	Complete loss of integrity	0
				Some loss of integrity (loosening of the protein fibers, holes)	1
				Integrity maintained (compact protein wall, protein fibers well organized)	2
New Quantitative assessment	Scaffan/HE staining	40× (Whole slide scan)	Structure length per area (mm/mm^2^)	<20	0
				20–60	1
				>60	2
			Number of branches per mm^2^	<10,000	0
				10,000–30,000	1
				>30,000	2

Along with morphological and molecular characteristics, mechanical properties also play a significant role in hepatic homeostasis. Likewise, the viscoelastic properties of the scaffold are critical to the morphology, viability, and metabolic activities of the implanted cells in liver engineering. For example, stiff or floppy scaffolds and substrates resulted in decreased vitality, spreading, and albumin (ALB) expression in repopulated HepG2 cells and hepatocytes (You et al., 2013; Mattei et al., 2018). Thus, establishing comprehensive and integrated decellularization assessment criteria will improve the quality and clinical translation of decellularized liver scaffolds.

## 4 Recellularization

The decellularized scaffold must be repopulated with functional cells to execute various physiological functions. The most vital cells are hepatic parenchymal cells, hepatocytes; however, the long-term *in vitro* survival of hepatocytes remains challenging. In addition, loss of the typical morphology and liver-specific functions provide evidence that the prolonged culturing of hepatocytes results in de-differentiation. Nonetheless, different stem cells and endothelial cells that have been applied in recellularization show great potential.

### 4.1 Primary Hepatocyte

Healthy livers can regenerate rapidly to maintain their standard size and function after a hepatectomy of 70%. However, primary hepatocytes scarcely proliferate *in vitro*. Therefore, obtaining sufficient viable hepatocytes is the biggest impediment to using primary hepatocytes for recellularization. The primary hepatocytes in a decellularized liver scaffold, cultured in a dynamic perfusion system, have higher viability, function, and can most crucially, proliferate, when compared to those cultured under static conditions (Kojima et al., 2018; Yang et al., 2018; Debnath et al., 2020; Sassi et al., 2021). Nonetheless, the prolonged culturing of primary hepatocytes results in the onset of apoptosis and a persistent decline in the function of certain cells. For example, the synthetic and metabolic abilities of primary hepatocytes were severely diminished after 1 week of recellularization, although histologically viable hepatocytes could still be observed (Butter et al., 2017; Debnath et al., 2020). Fetal hepatocytes have strong proliferative capacity and are a promising cell source for recellularization because of the progenitor cells in the fetal liver (Gridelli et al., 2012; Ogiso et al., 2016). Proliferation markers of fetal hepatocytes are 45-fold higher than those of adult hepatocytes, and reach the level of immortalized HepG2 cells. However, their hepatic function is relatively limited, and their use has generated much ethical concern (Gridelli et al., 2012; Ogiso et al., 2016).

### 4.2 Stem Cells

#### 4.2.1 Embryonic Stem Cell

Embryonic stem cells (ESCs) are pluripotent cells that are derived from the blastocyst stage of early mammalian embryos and can differentiate into various cell types and are capable of self-renewal (Damdimopoulou et al., 2016; Zhao H. et al., 2020). Under certain culture conditions, ESCs can differentiate into functional hepatocytes, cholangiocytes, and endothelial cells. Therefore, they are a promising source of cells for liver engineering (Touboul et al., 2010; Dianat et al., 2014; Lorvellec et al., 2017; Zhao H. et al., 2020). In contrast with static cultures, ESCs differentiate more efficiently in the liver scaffold, expressing less of the fetal hepatocyte marker, alpha fetoprotein (AFP), and more of the adult hepatocyte indicators, ALB, and lipid, as well as increased production of glycogen; however, ECSs have only half the level of ALB expressed in induced pluripotent stem cells (iPSCs) (Lorvellec et al., 2017). The primary concern for implanting ESCs into a scaffold is tumorigenicity, namely the development of teratoma and teratocarcinoma (Ben-David and Benvenisty, 2011; Damdimopoulou et al., 2016). The primary strategies for improving the safety of ESCs include ensuring ultimate differentiation, eliminating remnant pluripotent stem cells, interfering with the genes associated with tumor progression, and routine post-transplant monitoring (Ben-David and Benvenisty, 2011; Damdimopoulou et al., 2016).

Furthermore, ESCs research is ethically controversial because the extraction of ESCs necessitates the destruction of embryos (Damdimopoulou et al., 2016). It has instead been proposed that ESCs can be obtained from surplus embryos that are used for *in vitro* fertilization with the consent of the donor (Damdimopoulou et al., 2016). It is also feasible to isolate ESCs from cloned embryos using donor eggs and patient cells, and even individual blastocyst cells without harming the embryos (Rodin et al., 2014; Damdimopoulou et al., 2016).

#### 4.2.2 Induced Pluripotent Stem Cells

IPSCs can be obtained from adult tissue cells, where each pluripotent stem cell line is unique and would not cause an immune rejection response (Ben-David and Benvenisty, 2011; Vasylovska et al., 2020). IPSCs and derived hepatocyte-like cells (iPSC-HLCs) are likely cell sources for liver engineering. For example, human iPSC-HLCs and rat decellularized liver scaffolds were used to develop an artificial liver that could express ALB and CYP3A4. The new liver exhibited characteristic hepatic functions, such as protein synthesis and drug metabolism (Minami et al., 2019; Lorvellec et al., 2020). Nonetheless, AFP was still expressed in the recellularized liver, and the level of ALB in the recellularized liver was approximately 1/5 that of human iPSC-HLCs *in vitro* and 1/100 that of the recellularized liver with adult rat primary hepatocytes (Minami et al., 2019; Lorvellec et al., 2020). These results indicate that the HiPSC-HLCs in the *de novo* liver were immature, and induction and isolation of the HiPSC-HLCs caused cellular damage and reduced the functioning of hepatocytes. Takeishi implanted human iPSC-derived hepatocytes, endothelial cells, and cholangiocytes into rat liver scaffolds to generate a mini liver, which achieved 75% coverage of the liver vasculature and 66% coverage of the bile ducts (Takeishi et al., 2020). The human iPSC-derived mini liver had cell-cell and cell-ECM molecules, and functioned in immunocompromised rats for 4 days after auxiliary liver transplantation (Takeishi et al., 2020). Therefore, it is feasible to create a patient-specific bioengineered liver without immunogenicity. Additionally, the tumorigenic properties of iPSCs are roughly comparable to those of ESCs, and even iPSCs are more tumorigenic than ESCs because of genetic and epigenetic reasons (Ben-David and Benvenisty, 2011).

#### 4.2.3 Mesenchymal Stem Cells

Mesenchymal stem cells (MSCs) are multipotent stem cells with high proliferative capacity for self-renewal and can differentiate into the cell lines of the resident tissues (Li et al., 2017; Alfaifi et al., 2018; Kang et al., 2020). MSCs were initially identified and isolated from bone marrow. However, this approach requires invasive procedures, yields limited cell numbers, and the differentiation ability declines with aging. MSCs have also been isolated from the umbilical cord, adipose tissue, placenta, synovial membrane, and amniotic fluid (Alfaifi et al., 2018; Kang et al., 2020). The umbilical cord is the ideal source of MSCs because these cells are easily accessible and are more primitive, abundant, and proliferative than cells from other tissues (Alfaifi et al., 2018). Adipose tissue is also an alternative source of MSCs, where these cells have high proliferative capacity and are easily obtained by adiposuction (Kang et al., 2020). For example, after cultivation with human umbilical MSCs, a porcine liver scaffold expressed liver-specific proteins ALB, CK-18, and glycogen, and successfully differentiated from the hUC-MSC phenotype to the primary human hepatocyte phenotype (Li et al., 2017). More importantly, unlike ESCs and iPSCs, MCSs are devoid of ethical and tumorigenic issues, making them potential sources of cells for liver engineering (Xiang et al., 2016; Li et al., 2017).

### 4.3 Endothelial Cells

The daedal vasculature system of the liver is indispensable in metabolite transport and homeostasis. Therefore, endothelialization of the decellularized scaffold is crucial for successfully reconstructing a bioengineered liver. Currently, human umbilical vein endothelial cells (HUVECs) and LSECs are the most frequently used endothelial cells. To create an ideal whole liver scaffold, Verstegen et al. decellularized human discarded liver and demonstrated that implanting HUVECs into the scaffold effectively rebuilt the endothelial system (Verstegen et al., 2017). Similarly, recellularized livers with HUVECs, which were implanted in immunosuppressed pigs, supported perfusion for up to 15 days (Shaheen et al., 2020). Instillation of LSECs in rat decellularized livers forms an endothelial layer that reduces platelet deposition and improves the function of the co-implanted hepatocytes (Kojima et al., 2018).

The vasculature in the liver has a hierarchy, which involves the hepatic sinusoids to the larger vessels. However, most studies on revascularization have focused on larger vessels, instead of the microvasculature in the hepatic sinusoids. The absence of basement membrane resulted in severe damage to the microstructure of the hepatic sinusoids during decellularization. As previously mentioned (Section 2.1), the hepatic sinusoids and space of Disse are the sites of substance exchange between the liver and the blood, making microvascular revascularization at the sinusoids top-drawer to the new liver function. Watanabe et al. successfully used HUVECs coated with fibronectin and flow-induced mechanical stimulation to reconstruct the microvessels of the hepatic sinusoids (Watanabe et al., 2019). However, HUVECs and LSECs have distinct cell surface markers and permeability as aforementioned (Section 2.1), and LSECs are the preferred cells for re-endothelialization (Poisson et al., 2017; Shetty et al., 2018).

### 4.4 Combination

Several cells collaboratively accomplish hepatic functions; therefore, the liver cannot be recellularized with only 1 cell type to perform complicated hepatic functions. To generate a competent liver, the decellularized scaffold must be methodically infused with various cells, and these cells grow in the right site. A combination of the HA, PV, vena cava, and common bile ducts can be used for recellularlization. The common bile duct guides the hepatocytes to their anatomical sites, and re-endothelialization of the HA or PV can prevent thrombosis and guarantee sufficient blood perfusion to the rebuilt liver.

Cholangiocytes and hepatocytes that are perfused into the liver *via* the common bile duct and PV, respectively, can rebuild bile duct-like structures, while hepatocytes can settle in the liver parenchyma (Chen et al., 2019). In addition, hepatocytes and LSECs can be distributed in the hepatic parenchymal region and PV lumen after perfusion *via* the common bile duct and PV, respectively (Kojima et al., 2018). Takeishi demonstrated that the implantation of human iPSC-derived hepatocytes, endothelial cells, and cholangiocytes could cover the liver vasculature and bile ducts of decellularized rat liver (Takeishi et al., 2020). Anderson et al. implanted primary porcine hepatocytes into liver scaffolds previously reendothelialized with HUVECs could achieve ALB production, ammonia detoxification, and urea synthesis. Additionally, they found that the ectopic transplantation of engineered livers into pigs that have undergone a portocaval shunt can slow the accumulation of ammonia in the blood (Anderson et al., 2021). Kakabadze et al. discovered that the perfusion of liver tissue fragments that contained all cell types successfully hepatized decellularized placenta, resulting in the expression of ALB and glycogen (Kakabadze et al., 2018). The histological results also revealed that the hepatic cord and hepatic sinusoids were lined with endothelial cells, Kupffer cells, and other types of cells. The animal experimental results also indicated that the implantation of a hepatized placenta could prevent acute liver failure caused by excessive hepatectomy (Kakabadze et al., 2018). Multi-step perfusion and pressure control have also been shown to improve the implantation efficiency, cellular function, and microvascular formation in hepatic sinusoids (Soto-Gutierrez et al., 2011; Watanabe et al., 2019; Anderson et al., 2021).

## 5 Main Obstacles

### 5.1 Thrombogenicity

**FIGURE 3:**
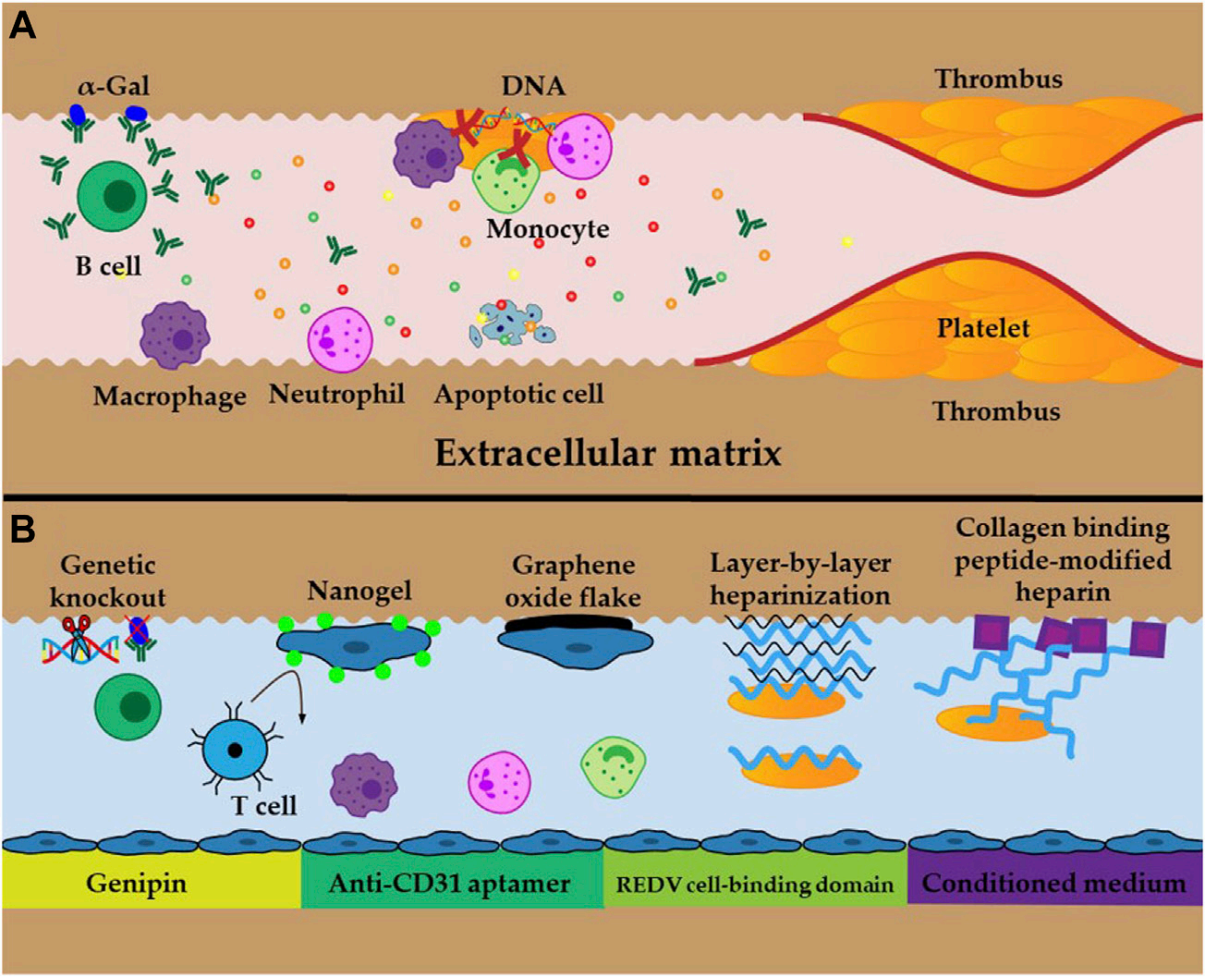
Schematic illustration of obstacles and approaches **(A)** Inflammatory response, oxidative stress, cell apoptosis, and thrombosis caused by residual DNA and Gal epitopes and exposed ECM **(B)** Examples of cross-linking and techniques to improve immunogenicity and thrombosis.

The primary advantage of whole decellularized liver scaffolds is that the intact vascular network is preserved. However, this advantage is at the expense of losing the endothelial layer (Shaheen et al., 2020). In the absence of an endothelial layer, the exposed components of the ECM, especially collagen, trigger platelet activation and aggregation, and generate thrombin and fibrin, which initiates the formation of a thrombus (Figure 3A). Additionally, residual DNA may activate platelets and exert significant pro-inflammatory and pro-thrombotic effects in the extracellular space, hence promoting the development of immunothrombosis (Fuchs et al., 2010; Shi et al., 2020). The resultant thrombosis contributes to an inadequate supply of nutrients and oxygen to the implanted cells, which impedes the long-term survival of the neo-liver (Sassi et al., 2021). Thus, re-establishing blood flow to the new liver entails inhibiting thrombosis and constructing a new endothelial layer. Heparin is the most prevalent anticoagulant and is extensively used in the lining of grafts to improve their hemocompatibility (Figure 3B) (Wu et al., 2021). Both *in vivo* and *in vitro* studies have shown that the layer-by-layer deposition of heparin onto decellularized scaffolds immobilizes heparin, which effectively prevents thrombosis, and neither recellularization nor the function of the hepatocytes is affected (Bruinsma BG et al., 2015; Liu et al., 2019). Jiang reported an easy-to-implement method for covalently binding collagen-binding peptides to heparin, where the modified heparin was selectively bound to collagen and dramatically reduced the adhesion of platelets and formation of thrombi (Jiang et al., 2016). Furthermore, scaffolds that were recellularized with HUVECs maintained stable blood perfusion under physiological stress when ectopically implanted in immunosuppressed pigs (Shaheen et al., 2020; Anderson et al., 2021). However, no study to date has achieved long-term *in vivo* perfusion because of imperfect revascularization, which remains a critical problem that must be addressed.

### 5.2 Anoikis

Anoikis is a particular type of apoptosis that results from the disruption of cell adhesion to the ECM (Chiarugi and Giannoni, 2008). *Ex vivo* perfusion and restoration of blood flow after transplantation generate reactive oxygen species (ROS) that hamper cell adhesion to the ECM and induce anoikis (Figure 3A), leading to low engraftment and survival of the implanted cells (Park et al., 2015). In addition, oxidative stress and inflammation suppress cell proliferation and enhance cellular senescence and death, which further reduce the success of the engrafted cells (Panahi et al., 2020). Therefore, it has been proposed that the transplant should be supplemented with antioxidants to scavenge ROS to increase the tolerance of the implanted cells to apoptosis. In addition, cross-linking approaches, such as the use of nanogels, graphene oxide sheets, anti-CD31 inducers, conditioned media, and REDV cell binding domains, also significantly improve cell adhesion to the ECM and increase engraftment in the implant (Figure 3B) (Park et al., 2015; Tang et al., 2017; Devalliere et al., 2018; Caires-Junior et al., 2021; Kim et al., 2021). These approaches are promising for the long-term survival of bioengineered livers because they increase cell engraftment and survival rates, which accelerate and improve vasculature reconstruction.

### 5.3 Immunogenicity

The prerequisite for the long-term *in vivo* survival of recellularized livers is low immunogenicity, which can be determined from the critical indicators of xenoantigenic residues (Figure 3A), such as residual DNA and galactose-a1,3-galactose (a-Gal) (Liu et al., 2018; Stahl et al., 2018). Reducing residual DNA and a-Gal involves improving the decellularization protocol and genetic knockout of a-Gal; these are proven approaches that diminish the immune response after implantation (Figure 3B) (Liu et al., 2018; Stahl et al., 2018). When sections of the liver scaffold were implanted subcutaneously in live animals, no immunogenicity was observed after 3 weeks, and there was low immunogenicity after 10 weeks (Mirmalek-Sani et al., 2013). Conversely, significant lymphocytic infiltration was observed 1 week after implantation, whereas at 3 weeks, the graft was almost completely degraded, and therefore, the inflammatory response was significantly reduced (Wang et al., 2016). Cross-linking the decellularized scaffolds with genipin and glutaraldehyde could minimize exposure of the immune cells to the ECM and increase cell adhesion of the implanted cells to the ECM (Figure 3B). When this approach is combined with immunosuppressive therapy, it can further improve the biocompatibility and long-term survival of the graft (Wang et al., 2016). Compared with glutaraldehyde, the genipin-crosslinked liver ECM exhibited superior biocompatibility and mechanical characteristics, and the *in vivo* immune responses of the host were lower (Gao et al., 2019). The graft from recellularized whole livers that was transplanted into living pigs was lost within 3 days without immunosuppression. Comparatively, this time period increased to more than 15 days when an immunosuppressive regimen was applied to the decellularized liver, and can potentially last longer if the immunosuppressive treatment is sustained (Shaheen et al., 2020). Therefore, more research into the concrete mechanism of immune rejection and strategies for suppressing immune rejection are critical for the long-term survival of bioengineered livers.

### 5.4 Shortage of Human Liver Scaffolds

Due to the shortage of discarded human livers, some researchers have proposed to populate human cells into the decellularized animal liver scaffolds to mimic human livers for transplantation. Xenotransplantation of genetically modified pig organs has attracted significant attention in recent years, particularly following the recent transplantation of a genetically modified pig kidney into a brain-dead human (Lu et al., 2019; Cooper, 2021). In contrast to the unsatisfactory microstructure of discarded human livers, animal livers are ideal and readily available. Human cells can colonize, proliferate, differentiate, and express ALB and glycogen in animal liver scaffolds (Li et al., 2017; Shaheen et al., 2020). However, the use of xenogeneic liver in human transplantation raises concerns due to the presence of the a-Gal. It has been shown that decellularized a-Gal knockout pig tissues behaved comparably to decellularized wild-type pig tissues (Stahl et al., 2018). Removing the a-Gal gene epitope reduced the adverse immune response of the recipient associated with prolonged exposure to decellularized xenografts (Stahl et al., 2018). Nevertheless, substantial concern has been expressed regarding the possibility of transmitting porcine endogenous retroviruses to the recipient and caregivers (Cooper, 2021).

Because of the risk of transmitting zoonosis, and immunological rejection of the xenogeneic liver, it is worthwhile to seek alternative human organs to replace the liver scaffold. The spleen and placenta are suitable alternative sources of scaffolds for bioengineered livers because they are readily available, have a vascular network for arterial and venous blood exchange, and contain ECM with diverse growth factors that support cell implantation (Maltepe and Fisher, 2015; Xiang et al., 2015; Liu et al., 2019). Decellularized spleen and placenta that are repopulated with primary hepatocytes and fresh liver fragments showed a characteristic hepatic cord structure, and expressed notable levels of ALB, urea, and glycogen after their ectopic transplantation into rats and sheep (Kakabadze et al., 2018; Liu et al., 2019). Moreover, this approach can mitigate against acute liver failure caused by extensive hepatectomy and promote regeneration of the original impaired liver. Consequently, other organs, such as the spleen and placenta, should be further explored as options to diversify the supply of organs for liver engineering (Kakabadze et al., 2018).

## 6 Conclusion and Prospects

This review discusses the structure of liver and hepatic ECM, and highlights the recent advances in decellularization and recellularization of the liver. An evaluation of decellularized scaffolds and obstacles is also presented. Although scaffold-based liver is a promising option for addressing the scarcity of donor liver, there are many limitations to the practical application of decellularized scaffolds: 1) ineluctable damage and denaturation of ECM proteins during decellularization, which hinders adhesion and proliferation of implanted cells; 2) imperfect revascularization, which lowers the hemocompatibility of the scaffold and increases the risk of thrombosis; and 3) elusory immune response, which causes inflammatory and rejection responses, resulting in loss of the graft. Future research that concentrates on these limitations will be valuable. Additionally, a better understanding of decellularization and recellularization of the liver is fundamental for producing transplantable liver grafts and ensuring the long-term survival of these grafts, especially if these insights are coupled with a deeper understanding of the mechanisms of the immune response.
